# A Subspace Method for Dynamical Estimation of Evoked Potentials

**DOI:** 10.1155/2007/61916

**Published:** 2007-11-12

**Authors:** Stefanos D. Georgiadis, Perttu O. Ranta-aho, Mika P. Tarvainen, Pasi A. Karjalainen

**Affiliations:** Department of Physics, University of Kuopio, P.O. Box 1627, 70211 Kuopio, Finland

## Abstract

It is a challenge in evoked potential (EP) analysis to incorporate prior physiological knowledge for estimation. In this paper, we address the problem of single-channel trial-to-trial EP characteristics estimation. Prior information about phase-locked properties of the EPs is assesed by means of estimated signal subspace and eigenvalue decomposition. Then for those situations that dynamic fluctuations from stimulus-to-stimulus could be expected, prior information can be exploited by means of state-space modeling and recursive Bayesian mean square estimation methods (Kalman filtering and smoothing). We demonstrate that a few dominant eigenvectors of the data correlation matrix are able to model trend-like changes of some component of the EPs, and that Kalman smoother algorithm is to be preferred in terms of better tracking capabilities and mean square error reduction. We also demonstrate the effect of strong artifacts, particularly eye blinks, on the quality of the signal subspace and EP estimates by means of independent component analysis applied as a prepossessing step on the multichannel measurements.

## 1. INTRODUCTION

Evoked potentials (EPs) and ongoing brain activity oscillations, obtained by scalp electroencephalogram (EEG) recordings, have been linked with various cognitive processes and provide means for studying cerebral brain function [1]. An EP is usually considered to be a wave or complex elicited by and time-locked to a physiological or nonphysiological stimulation or event. EPs are buried into background brain activity, and nonneural activity like muscle noise. Since many parallel mental processes may occur simultaneously in the brain, it is difficult to observe and determine an evoked potential on a single-trial base. Therefore, the simplest way to investigate EPs is to use ensemble averages of time-locked EEG epochs obtained by repeated stimulation. It is well known that this signal enhancement implies a loss of information related to trial-to-trial variability, and nonstationary features of event-related phenomena.

The generation mechanism of evoked responses is not precisely known in many situations. EPs are assumed to be generated either separately of ongoing brain activity, or through stimulus-induced reorganization of ongoing activity. For example, it might be possible that during the performance of an auditory oddball discrimination task, the brain activity is being restructured while attention is focused on the target stimulus [2]. Phase synchronization of ongoing brain activity is one possible mechanism for the generation of event-related responses. That is, following the onset of a sensory stimulus, the phase distribution of ongoing activity changes from uniform to one which is centered around a specific phase [3]. Moreover, several studies have concluded that averaged EPs are not separate from ongoing cortical processes, but rather, are generated by phase synchronization and partial phase resetting of ongoing activity [4, 5]. However, phase coherence over trials observed with common signal decomposition methods (e.g., wavelets) can result both from a phase-coherent state of ongoing rhythms and from the presence of a phase-coherent event related potential, which is additive to ongoing EEG [6]. Furthermore, stochastic changes in amplitude and latency of different components of the EPs are able to explain significant part of intertrial variability of the measurements [6–9].

Several methods have been proposed for EP estimation and denoising; see, for example, [10–13]. In general, most of the methods for single-trial EP analysis aim to decompose the measurements into relevant components or to explain the data through some parameters. The parametrization gives the necessary means to investigate, for example, the changes that the stimulus causes to the ongoing EEG signal, or that the repetition of the test causes to the responses. Most of the methods are based on an explicit model or on some specific assumptions for the EPs. Every decomposition then involves at least two main considerations. On the one hand, if the resulting estimates follow too closely the measurements, it is possible that some features of the data are still going to be hidden by phenomena unrelated to the stimulation. On the other hand, if the estimates do not follow the measurements, some features may have been neglected. Usually a balance between these considerations is made and care is given to the correct interpretation of a parametrization that is able to reveal specific features of the experiment.

The performance and applicability of every single-trial estimation method depends on the prior information used and the statistical properties of the EP signals. Here, we focus on the case that some parameters of the EPs change dynamically from stimulus to stimulus. This situation could be a trend-like change of the amplitude or latency of some phase-locked component of the EPs. Although, for example, the above-mentioned methods [10–13] could be used to estimate such changes, they do not take into account in the estimation procedure this trend-like variability.

The most obvious way to handle time variations between single-trial measurements is subaveraging of the measurements in groups. Subaveraging could give optimal estimators if the EPs are assumed to be invariant within the subaveraged groups. A better approach is to use moving window or exponentially weighted average filters; see, for example, [14, 15]. Other adaptive methods have also been proposed for EP estimation, especially for brain stem potential tracking, for example, [16]. The statistical properties of some average filters and different recursive estimation methods for EP estimation have been discussed through Kalman filtering in [17]. Some smoothing methods have also been proposed for modeling trial-to-trial variability in EPs (e.g., [18]).

An elegant way to describe trial-to-trial variations in EPs can be given through state-space models. State-space modeling for single-trial dynamical estimation considers the EP as a vector-valued random process with stochastic fluctuations from stimulus to stimulus [17]. Then, past and future realizations contain information of relevance to be used in the estimation procedure. Recursive estimates for the states, that are optimal in the mean square sense, are given by Kalman filter and smoother algorithms. Of importance is also the parametrization of the problem and the selection of an observation model for the measurements. For example, in [16, 17] generic observation models were used based on shifted Gaussian-shaped smooth functions. While other generic observation models could also be considered, when all the measurements are available, data-based observation models can be used.

In this paper, we extend the method presented in [17] to the use of Kalman smoother algorithm. We demonstrate that for batch processing the use of the smoother algorithm is preferable. Fixed-interval smoothing improves the tracking performance of EP characteristics and reduces greater the noise. In parallel, we propose a novel method for state-space modeling of EPs. The method is based on the eigenvalue decomposition of the ensemble data correlation matrix. A few dominant eigenvectors form a signal subspace that can be used for single-trial estimation. Subspace-based methods have already been proposed for EP estimation, for example, in [12, 19]. However, these approaches do not take into account in the estimation procedure the situation that some characteristics of the EPs change dynamically from stimulus to stimulus. In this paper, we demonstrate that such a signal subspace can be used to model dynamic changes present in EP measurements.

The approach is demonstrated with simulated and real measurements obtained by an auditory EP experiment. Finally, we investigate the effect of strong artifacts on the quality of the estimates by means of independent component analysis (ICA), which is applied as a prepossessing step on the multichannel measurements.

## 2. METHODS

The sampled potential (from channel l) relative to the successive stimulus or trial t can be denoted with a column vector of length M:

(1) $$z_{t}=\left(\begin{array}{c} z_{t}(1)\\ z_{t}(2)\\ \vdots \\ z_{t}(M) \end{array}\right),\;t=1,\ldots ,T,$$

where T is the total number of trials.

### 2.1. Linear estimation and additive noise model

A widely used model for EP estimation is the additive noise model. The observations are then assumed to be of the form

(2) $$z_{t}=s_{t}+\upsilon_{t}.$$

The vector $s_{t}$ corresponds to the part of the activity that is related to the stimulation, and the rest of the activity $\upsilon_{t}$ is usually assumed to be independent of the stimulus and the EP. Single-trial EPs can be further modeled as a linear combination of some preselected basis vectors. Then, the observation model takes the form

(3) $$z_{t}=H_{t}\theta_{t}+\upsilon_{t},$$

where $H_{t}$ is the observation matrix, which contains the basis vectors $\psi_{t,1},\ldots ,\psi_{t,k}$ of length M in its columns, and $\theta_{t}$ is a parameter vector of length k. The estimated EPs ${\hat{s}}_{t}$ can then be obtained by using the estimated parameters ${\hat{\theta }}_{t}$ as follows:

(4) $${\hat{s}}_{t}=H_{t}{\hat{\theta }}_{t}.$$

By treating both $\theta_{t}$ and $\upsilon_{t}$ as random, the estimator ${\hat{\theta }}_{t}$ that minimizes the mean square Bayes cost BMS=E{∥θt−θ^t∥2} is given by the conditional mean [20]

(5) θ^t=E{θt∣zt}

of the posterior distribution

(6) $$\begin{array}{ll} p(\theta_{t}|z_{t}) & \propto p(z_{t}|\theta_{t})p(\theta_{t})\\ & \propto p_{\upsilon_{t}}(z_{t}-H_{t}\theta_{t}|\theta_{t})p(\theta_{t}). \end{array}$$

By taking into account the linear observation model, and that $\theta_{t}$ and $\upsilon_{t}$ are assumed uncorrelated, that is, $C_{\theta_{t},\upsilon_{t}}=0$. the linear conditional mean estimator takes the form [20]

(7) θ^t=(HtTCυt−1Ht+Cθt−1)−1(HtTCυt−1zt+Cθt−1ηθt),

where $C_{\theta_{t}}$ and $\eta_{\theta_{t}}$ are, respectively, the covariance and the mean of $\theta_{t}$. $C_{\upsilon_{t}}$ is the covariance of the zero mean measurement noise, and ${\left(\cdot \right)}^{T}$ denotes transpose. The estimator is optimal in the mean square sense among all possible estimators, not only linear, if $\theta_{t}$ and $\upsilon_{t}$ are Gaussian. In Bayesian estimation this is also called the maximum a posteriori estimator (MAP), and $C_{\theta_{t}}$ and $\eta_{\theta_{t}}$ represent prior information about the parameters $\theta_{t}$. If they are not available, we can assume $C_{\theta_{t}}^{-1}=0$ corresponding to infinite prior variance for the parameters. In this case, the estimator reduces to the ordinary minimum variance Gauss-Markov estimator, which treats the parameters as nonrandom. If we assume that the errors are independent with equal variances $C_{\upsilon_{t}}=\sigma_{\upsilon_{t}}^{2}I$. the estimator is identical to the ordinary least squares estimator

(8) $${\hat{\theta }}_{t}=(H_{t}^{T}H_{t}{)}^{-1}H_{t}^{T}z_{t}.$$

### 2.2. State-space modeling of EPs

Estimators of the form (7) can be used to model time-varying characteristics of EPs, for example, in terms of amplitude and latency estimates of some characteristic peak of the signals. However, such estimators do not take into account situations that some dynamical behavior is expected from stimulus to stimulus. A mathematical plausible way to incorporate prior information for estimation about time-varying phenomena is given through state-space modeling.

The measurement vectors $z_{t}$ can be considered as realizations of a stochastic vector process, that depends on some unobserved parameters $\theta_{t}$ (state vector) through the model (3). The parameters $\theta_{t}$ are the quantities that we are primarily interested in, and their form depends on the parametrization of the estimation problem. In order to model the time evolution of the hidden process $\theta_{t}$, a linear first-order Markov model can be used, that is,

(9) $$\theta_{t}=F_{t}\theta_{t-1}+\omega_{t},$$

with some initial distribution for $\theta_{0}$. Equations (3) and (9) form a linear state-space model, where $F_{t}$ and $H_{t}$ are preselected matrices. Other important assumptions for the model are

- for every i≠j, the observation noise vectors $\upsilon_{i},\;\upsilon_{j}$ as well as the state noise vectors $\omega_{i},\;\omega_{j}$ are mutually independent and also mutually independent of the initial state $\theta_{0}$,
- the vectors $\omega_{i},\;\upsilon_{j}$ are mutually independent for all i, j.

For the white noise sequences $\omega_{t}$ and $\upsilon_{t}$, we can also assume $E\{\omega_{t}\}=0$ and $E\{\upsilon_{t}\}=0$ for every t, but the covariances $C_{\omega_{t}}$, $C_{\upsilon_{t}}$ can still be time-varying.

### 2.3. Kalman filter and smoother algorithms

The Kalman filtering problem is related to the determination of the mean square estimator ${\hat{\theta }}_{t}$ for the state $\theta_{t}$ given the observations $z_{1},\ldots ,z_{t}$. This is equal to the conditional mean

(10) θ^t=E{θt∣z1,…,zt}=E{θt∣Zt},

that relates to the density [20]

(11) $$p(\theta_{t}|Z_{t})\propto p(z_{t}|\theta_{t})p(\theta_{t}|Z_{t-1}),$$

where

(12) $$p(\theta_{t}|Z_{t-1})=\int p(\theta_{t}|\theta_{t-1})p(\theta_{t-1}|Z_{t-1})d\theta_{t-1}.$$

The optimal linear mean square estimator can then be obtained recursively by restricting to a linear conditional mean, or by assuming $\upsilon_{t}$ and $\omega_{t}$ to be Gaussian [20]. The recursive estimator can be written as

(13) θ^t=(HtTCυt−1Ht+Cθ˜t∣t−1−1)−1(HtTCυt−1zt+Cθ˜t∣t−1−1θ^t∣t−1), 

where θ^t∣t−1 is the prediction of $\theta_{t}$ based on ${\hat{\theta }}_{t-1}$ and θ^t−1=E{θt−1∣zt−1,…,z1} is the optimal MS estimate at time t−1. Clearly this is of the form (7), which is the Bayesian MAP estimator using the last available estimate as prior information. After adding the initializations, Kalman filter algorithm can be written as follows.

- Initialization:
  (14) Cθ˜0=Cθ0,θ^0=E{θ0}.
- Prediction step:
  (15) θ^t∣t−1=Ftθ^t−1,Cθ˜t∣t−1=FtCθ˜t−1FtT+Cωt.
- Filtering step:
  (16) Kt=Cθ˜t∣t−1HtT(HtCθ˜t∣t−1HtT+Cυt)−1,θ^t=θ^t∣t−1+Kt(zt−Htθ^t∣t−1),Cθ˜t=(I−KtHt)Cθ˜t∣t−1,

for t=1,…,T. The matrix $K_{t}$ is called the Kalman gain matrix.

If all the measurements are available, that is, $z_{t},\;t=1,\ldots ,T$, then the fixed interval smoothing problem can be considered, that is,

(17) θ^ts=E{θt∣z1,…,zT}=E{θt∣ZT},

that relates to the density [21]

(18) $$p(\theta_{t}|Z_{T})=p(\theta_{t}|Z_{t})\int \frac{p(\theta_{t+1}|\theta_{t})p(\theta_{t+1}|Z_{T})}{p(\theta_{t+1}|Z_{t})}d\theta_{t+1}.$$

The last form suggests again a recursive estimation procedure for the determination of the conditional density. It is thus possible to compute filtered and prediction distributions in a forward (filtering) recursion, and then execute a backward recursion with each smoothed distribution $p(\theta_{t}|Z_{T})$ relying upon the quantities calculated in the forward run and the previous (in reverse time) smoothed distributions $p(\theta_{t+1}|Z_{T})$. This property enables the formulation of the forward-backward method for the smoothing problem [20], which gives the smoother estimates as corrections of the filter estimates. So for the linear or Gaussian case the smoothing problem is complete through the backward recursion.

Smoothing:

(19) At=Cθ˜tFt+1TCθ˜t+1∣t,θ^ts=θ^t+At(θ^t+1s−θ^t+1∣t),Cθ˜ts=Cθ˜t+At(Cθ˜t+1s−Cθ˜t+1∣t)AtT,

for t=T−1, T−2,…,1. For the initialization of the backward recursion the filter estimates can be used, that is, ${\hat{\theta }}_{T}^{s}={\hat{\theta }}_{T}$.

### 2.4. Signal and noise subspaces

Singular value decomposition (SVD) has many theoretical and practical applications in signal processing and identification problems [23]. In relatively high signal-to-noise ratio conditions (SNR), SVD of a data matrix can divide measurements into signal and noise subspaces. Alternatively, it can also be understood in terms of principal component regression (PCR) as a combined method for signal enhancement and optimal model dimension reduction [24]. The subspace method has been used to enhance stimulus phase-locked activity in different studies (e.g., [19]).

The available data matrix $Z=[z_{1},\ldots ,z_{T}]\in {\mathbb{R}}^{M\times T}$, which has as columns the EEG sampled epochs relative to the stimulation, can be decomposed as

(20) $$Z=U\Sigma V^{T},$$

where $U\in {\mathbb{R}}^{M\times M}$ satisfies $U^{T}U=I$, $V\in {\mathbb{R}}^{T\times T}$ satisfies $V^{T}V=I$, and $\Sigma \in {\mathbb{R}}^{M\times T}$ is a pseudodiagonal matrix with nonnegative diagonal elements $\sigma_{i}$ such that $\sigma_{1}\geq \sigma_{2}\geq \cdots \geq \sigma_{\min(M,T)}\geq 0$. If M≤T, then Σ has the form $\Sigma =[\Sigma_{1},0]$, where $\Sigma_{1}=\text{diag}(\sigma_{1},\ldots ,\sigma_{M})$ and 0 is a zero matrix. If M>T, then Σ has the form $\Sigma =\left[\begin{array}{c} \Sigma_{1}\\ 0 \end{array}\right]$, where $\Sigma_{1}=\text{diag}(\sigma_{1},\ldots ,\sigma_{T})$. Only r singular values are nonzero, where r=rank(Z).

For the additive noise model and relatively small noise the following decomposition can be considered:

(21) $$Z=[U_{s},U_{\upsilon }]\left[\begin{array}{cc} \Sigma_{s} & 0\\ 0 & \Sigma_{\upsilon } \end{array}\right]{\left[V_{s},V_{\upsilon }\right]}^{T}.$$

The matrix $\Sigma_{s}$ contains the k largest singular values and $U_{s}$ the respective left singular vectors associated mainly with the signals $s_{t}$. Thus the matrices $\left(U_{s},\Sigma_{s},V_{s}\right)$ represent a signal subspace, and $\left(U_{\upsilon },\Sigma_{\upsilon },V_{\upsilon }\right)$ represent primarily the noise subspace.

From the SVD of the matrix $Z=U\Sigma V^{T}$ we also have

(22) $$ZZ^{T}=U\Sigma_{1}^{2}U^{T}.$$

This means that the left singular vectors of Z are the eigenvectors of the matrix $ZZ^{T}$, or the eigenvectors of the data correlation matrix

(23) $$\hat{R}=\frac{1}{M}ZZ^{T}.$$

If we denote with $H_{s}$ the matrix with columns the k dominant eigenvectors, then the ordinary least squares estimator for the parameters $\theta_{t}$ becomes

(24) θ^t=(HsTHs)−1HsTzt=HsTzt.

Estimates for the EPs can then be obtained from (4). Quantitatively, the first basis vector is the best mean-square fit of a single waveform to the entire set of epochs. Thus, the first eigenvector is similar to the mean of the epochs, and the corresponding parameters or principal component ${\hat{\theta }}_{t}(1)$ (t=1,2,…,T) reveal the contribution of the eigenvector to each epoch. The rest of the dominant eigenvectors model primarily amplitude differences between individual EP peak components, and latency variations from trial to trial. Therefore, since this basis contains prior information about phase-locked characteristics of the EP signals, we consider the following state-space model for dynamical estimation:

(25) $$\begin{array}{ll} \theta_{t} & =\theta_{t-1}+\omega_{t},\\ z_{t} & =H_{s}\theta_{t}+\upsilon_{t}, \end{array}$$

with the selections $F_{t}=I$, t=1,…,T, that is, a random walk model, and $H_{t}=H_{s}$ for all t. Estimates for the parameters can then be obtained by Kalman filter and smoother algorithms for different selections of state and observation noise covariance matrices. Thus, the applicability of the proposed method relates on the quality of the signal subspace in low signal-to-noise ratio conditions, as well as on the assumption of hidden dynamical behavior from trial-to-trial.

### 2.5. Artifact correction by ICA

Individual EEG channels measure superimposed activity generated simultaneously by various brain sources. The behavior of the sources is stochastic and generally nonstationary. In addition, artifact sources, such as eye blinks, can distort statistical properties of the signals and increase complexity. For the problem of blind source separation (BSS) of the multichannel EEG measurements, target is to recover unobserved brain generated initial source signals by using only the available sensor data and some statistical properties assumed for the sources [25, 26].

Fundamentally, the basic problem that BSS attempts to solve assumes a set of L measured data points $x_{n}=(x_{n}(1),\ldots ,x_{n}(l),\ldots ,x_{n}(L{))}^{T}$ at time instant n (n=1,…,N) to be a linear combination of m unknown sources $y_{n}=(y_{n}(1),\ldots ,y_{n}(m{))}^{T}$, that is

(26) $$x_{n}=Ay_{n}+\upsilon_{n}.$$

For EEG measurements, L is the number of available channels, and the measurements can be summarized in a matrix X having the vectors $x_{n}$ in its columns and different channel recordings in each row. A time-invariant mixing matrix A is the common approach for ICA and BSS of EEG, for example, in event-related studies [3]. This model can be interpreted as the fixed biophysical structure of the brain itself whilst the sources distributed within this structure change their intensity over time [25].

A general formulation for BSS without any assumptions (prior information) about the nature of the data, noise, or mixing system will leave the problem of EEG separation intractable. Therefore, some basic assumptions are needed. For example, the goal of ICA is to recover independent sources given only sensor observations that are unknown linear mixtures of unobserved independent source signals [27, 28].

The assumption of physiological independence of the sources can be quite obvious in some situations, for example, when used in artifact rejection separating brain signals from ocular artifacts. Note that the ICA model considers the signals as independent and identically distributed, and requires non-Gaussian sources. Thus, by ignoring time structure, the estimation is based solely on investigating structure across the sensors as estimated by the sample distribution of the measurements, and an embedded density parametrization (differentiating at least between sub-Gaussian and super-Gaussian sources). Therefore, the model might not be able to separate every kind of sources (e.g., stationary Gaussian random processes). However, in many situations predominant artifacts show a highly kurtotic sample distribution that enables estimation.

ICA methods carry ambiguities about the ordering and the overall amplitude and sign of the estimated sources. The rows of the data matrix X are the EEG channel recordings and are decomposed as X=AY, where Y has in its rows the independent components. The mixing matrix A contains the spatial information of the sources obtained at the sensors. Therefore, the columns of A are the spatial distributions of the estimated sources, which are normalized to unit variance. For example, eye movements and eye blinks project mainly to frontal sites. An artifact source can be eliminated and removed from the measurements by backprojection.

## 3. RESULTS

In this section, we present the performance of Kalman filter and smoother algorithms on tracking dynamic variations, and estimating single-trial EPs in a simulated and a real data set. In parallel, we investigate the performance of the method when the signal subspace is enhanced by rejecting eye-related artifacts with the use of ICA.

### 3.1. Measurements and artifact removal

EEG measurements were obtained from a standard oddball paradigm with auditory stimulation (1 subject, 60 EEG channels, reference: ears). In the recording, 569 auditory stimuli were presented with an interstimulus interval of 1 second. Eighty-five percent of the stimuli were the standard tones at 800 Hz. Fifteen percent were the deviant tones at 560 Hz. The deviant tones were randomly presented. The subject was sitting in a chair, and was asked to press a button every time he heard the deviant target tone. The sampling rate of the measurements was 500 Hz.

Reduction in noise for EEG signals can be done with linear filtering without altering the basic ICA model [27]. If we further assume less sources than sensors and that the sensor noise is relatively small, then principal component analysis (PCA) on the data covariance matrix and dimension reduction can be used to reduce the noise and to prevent overlearning [27]. For the analysis, the data were digitally filtered in the range (1–35 Hz). All the measurement set (about 10 minutes) was used for the estimation of the separating matrix. The dimension of the data was reduced with PCA to 31, by keeping eigenvectors associated with eigenvalues larger than 1, resulting in more than 99% of explained variance. The Fast ICA algorithm in parallel form [27] was used for the estimation of independent components.

**Figure 1:**
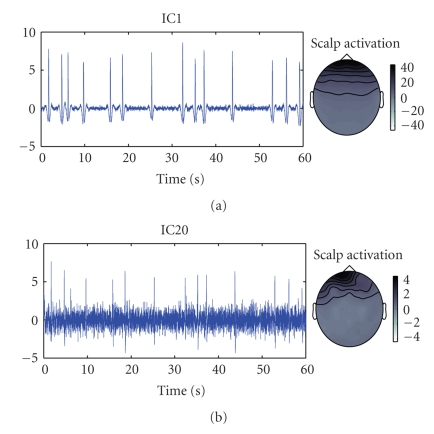
Blink-related components estimated with ICA. Time activations (left) and scalp activations (right). The left plots correspond to the first minute of the measurement set.

By visual inspection of the estimated components and scalp activations two components showed to be related to eye activity. The blink components are presented in Figure 1. On the left, the time activations corresponding to the first minute of the recordings are presented, and on the right the spatial distributions. Furthermore, these components did not show any significant correlation with the two types of stimuli (standard and target). Correlation with stimulation time was investigated by computing EP image plots for every estimated component. The component-based EP image plots are not shown here, but such images are also used in the next section (Figures 3 and 5). EP image plots are constructed by color-coding potential variations occurring in single-trial epoch vectors (e.g., [3]). The thin color-coded horizontal bars, each representing a single-trial, are, for example, stacked row-by-row according to data collection time (data epochs sampled relative to successive stimulus or trial t) producing an EP image.

Note that PCA-based dimension reduction is a rather subjective approach for the determination of the number of brain source signals in EEG measurements [25]. Some relatively weak brain sources, as measured at the sensors, may be eliminated. Additionally, some estimated independent components may remain the mixture of more than one source signals. However, by computing different EP image plots we did not observe any significant loss of phase-locked EP activity. Furthermore, filtering and dimension reduction provided good estimates for the blink components and fast convergence for the Fast ICA algorithm. Therefore, the performance was considered satisfactory for ocular artifact removal, and for the demonstration needs of the proposed subspace method for dynamical estimation of single-channel single-trial EPs.

### 3.2. Single-trial estimation

Real EEG data were used as background EEG activity, or noise, in the simulations. From the recordings, we used only the channel CZ, after preprocessing and artifact removal by ICA. Only ocular artifacts were considered. As background activity for the simulations, we sampled prestimulus EEG epochs from −500 milliseconds to 0 millisecond relative to the standard stimulus onset. Simulated EPs were constructed according to the additive noise model by superimposing upon the selected real EEG epochs linear combinations of 2 Gaussian-shaped functions. In order to be consistent to the real measurements (standard tones and N100/P200 complex), each pseudoreal EP vector has two Gaussian peaks: a negative after 100 milliseconds and a positive after 200 milliseconds. Trial-to-trial sinusoidal variations for the amplitude and latency of the second peak were generated. Random variations were also added to the amplitudes, latencies, and widths of both simulated peaks.

**Figure 2:**
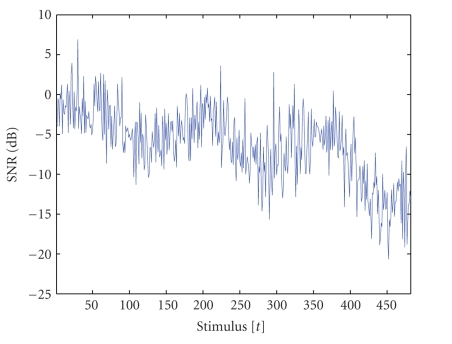
Time-varying SNR(dB) for the simulated second peak as a function of the stimulus number (trial) t, that is, ${\text{SNR}}_{t}=10{\;\log}_{10}{\;\sum }_{i}s_{t}^{2}(i)/\sum_{i}\upsilon_{t}^{2}(i)$, t=1,…,T, where $s_{t}$ are the simulated noise-free single-trial EPs and $\upsilon_{t}$ prestimulus EEG epochs sampled relative to the standard tone from channel CZ after ocular artifact removal with ICA. The sums were considered in a smaller interval around 200 milliseconds covering only the second peak (see also Figure 3).

**Figure 3:**
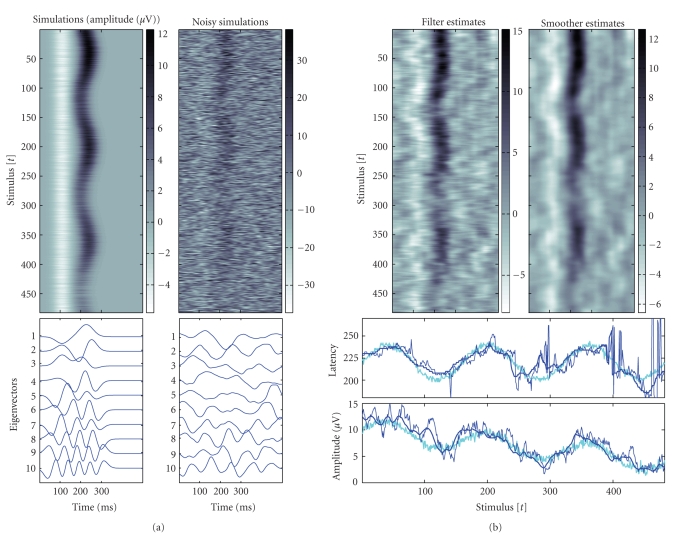
Simulations resembling the N100/P200 auditory complex and obtained estimates. For background noise prestimuli EEG samples relative to standard tones from channel CZ after ocular artifacts removal were used. Left: simulations (Gaussian functions) and noisy simulations, single-trials as image plots (up), and the respective 10 dominant eigenvectors of the data correlation matrix (bottom). The EP images represent stimulus locked stacked epochs (row-by-row). The color-maps describe the amplitude level in μV, y-axis represents successive stimulus or trial t, and the x-axis represents within a trial latency variation. Right: single-trial estimates as image plots with Kalman filter and smoother (up) and estimated variability of the second positive peak (bottom). Simulated amplitude and latency trends (light bold), estimates based on Kalman filter (dark thin) and based on fixed-interval Kalman smoother (dark bold). For estimation the selection $\sigma_{\omega }^{2}{=10}^{-2}$ was used.

The estimated time-varying SNR with respect only to the second peak can be seen in Figure 2 as a function of the stimulus number t. Therefore, the important assumption in the simulations is the trend-like behavior in low signal-to-noise ratio conditions. By construction the simulated EPs have trend-like trial-to-trial characteristics. This can be observed in Figure 3 (left) and the EP image plots. In the same figure (bottom, left), they are also presented the 10 dominant eigenvectors of the data correlation matrix obtained before and after EEG addition.

It must be noted that the aim in the creation of the simulations was that the average of the simulated EPs is close to the average of the real measurements (standard tones and N100/P200 complex at channel CZ). The average of the real measurements has a negative peak around 110 milliseconds (N100) with amplitude about −4 μV, and a positive peak (P200) around 230 milliseconds with amplitude about 5 μV. Then the simulations were created as follows. For the first peak random variability in a small range in amplitude and latency was simulated that gives ensemble average with peak amplitude about −4 μV at the required latency. For the second peak dynamic variability was created with range of about 10 μV (2–12 μV) in amplitude and about 45 milliseconds in latency, such that the average has peak amplitude about 6 μV and similar latency to the real measurements. Then prestimuli EEG was added. In that respect, SNR conditions were not directly considered, but instead a reasonable range for the time-varying behavior was assumed that can produce similar average with the real measurements.

For estimation the state-space model (25) was selected. For the covariances we used $C_{\omega_{t}}=\sigma_{\omega }^{2}I$ and $C_{\upsilon_{t}}=\sigma_{\upsilon }^{2}I$ for every stimulus t. Then the selection of the last variance term is not essential since only the ratio $\sigma_{\upsilon }^{2}/\sigma_{\omega }^{2}$ has effect on the estimates. Then the choice $C_{\upsilon_{t}}=I$ can be made and care should be given to the selection of $\sigma_{\omega }^{2}$. In general, if it is tuned too small, fast fluctuations of EPs are going to be lost, and if it is selected too big the estimates have too much variance and they will tend to be similar to the ordinary least squares or principal component regression solution. The selection can be based on experience and visual inspection of the estimates as a balance between preserving expected dynamic variability and greater noise reduction.

In order to identify an optimal value for the variance term $\sigma_{\omega }^{2}$ for the simulations we calculated root mean square errors (RMSEs) between the estimates based on the noisy data and the noiseless simulated EPs. The RMSEs were computed with respect to the second peak only over a smaller time interval around 200 milliseconds. For initialization of the algorithms we used half the data set by filtering backwards in time. The last estimates were used for initializing the forward run. Finally, the last state estimate of the Kalman filter forward run was used to initialize the backward smoothing procedure.

**Figure 4:**
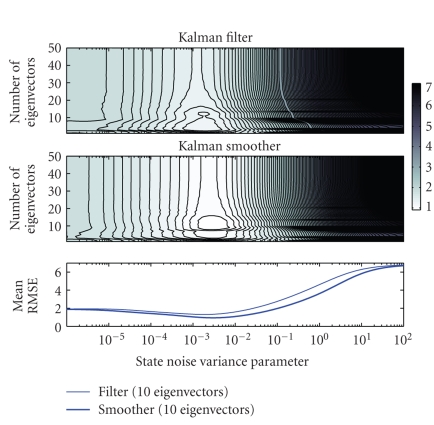
Means of RMSEs for different values of the state noise variance parameter $\sigma_{\omega }^{2}$ and different number of dominant eigenvectors included in the observation model. Contour plots of the means for Kalman filter (top) and smoother (middle). Means when 10 eigenvector are included in the observation model (bottom). In all plots the x-axis is in logarithmic scale.

Means of RMSEs over all single-trials for different values of state noise variance parameter and for different dimensions of the observation matrix are presented in Figure 4 as contour plots for Kalman filter (top) and smoother (middle). In all the cases, Kalman smoother provides smaller error than the filter. This is to be expected, since all the measurements are included in the estimation procedure. The reduction of the error during backward smoothing is due to greater noise cancellation, as well as better tracking of the dynamic fluctuations. Optimal values of $\sigma_{\omega }^{2}$ for all the selected observation matrices are between $10^{-3}$ and $10^{-2}$. By considering the contour plots and by inspection of the estimates, around 10 eigenvectors are enough for tracking the dynamic fluctuations. Single-trial estimates for that dimension (k=10) and with the selection $\sigma_{\omega }^{2}{=10}^{-2}$ are presented in Figure 3 as image plots for Kalman filter and smoother. In the right (bottom) of the same figure they are presented estimates for the single-trial latency and amplitude of the second peak as a function of the stimulus number or trial t.

State-space representation and a few dominant eigenvectors obtained from the ensemble data correlation matrix are able to model the amplitude and latency changes. Bayesian recursive mean square estimation is able to reveal the hidden dynamic variability under unfavorable signal-to-noise ratio conditions. Clearly, Kalman smoother tracks better the dynamic changes and reduces greater the noise.

**Figure 5:**
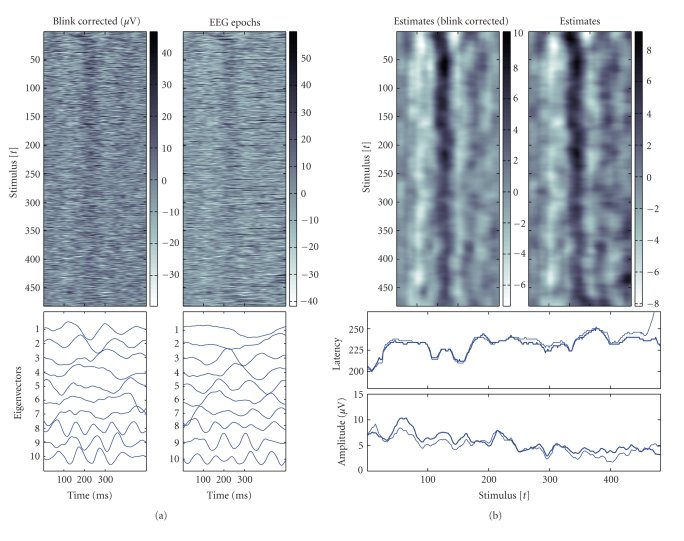
N100/P200 auditory complex, measurements from channel CZ. EEG epochs relative to the standard tones (0–500 milliseconds after auditory stimulation), and obtained estimates. Left: EEG epochs as image plots after and before blink correction (up) and the respective 10 dominant eigenvectors of the data correlation matrix (bottom). The EP images represent stimulus locked stacked epochs (row-by-row). The color-maps describe the amplitude level in μV, y-axis represent successive stimulus or trial t, and the x-axis within a trial latency variation. Right: single-trial estimates as image plots with Kalman smoother (up) based on artifact corrected measurements and original measurements respectively, amplitude and latency estimates of the P200 peak (bottom) based on original measurements (thin) and artifact corrected measurements (bold). For estimation the selection $\sigma_{\omega }^{2}{=10}^{-2}$ was used.

For the real measurements we considered epochs 0–500 milliseconds after the presentation of the standard tones from channel CZ before and after eye artifact removal. For the two data sets we selected 10 eigenvectors of the data correlation matrix for estimation. The strong blink contributions clearly affect the eigenvectors and the signal subspace, especially after the first half of the measurements, see Figure 5. This can also be seen by observing the first two eigenvectors that reflect mainly blink artifacts. However, since the blinks occur random enough, recursive mean square estimation is largely reducing their contribution. This can be observed in Figure 5 from the estimates, which are obtained with Kalman smoother with the same choices $\sigma_{\omega }^{2}{=10}^{-2}$ and k=10 for both data sets. The estimated dynamic variability of the second peak (P200) in terms of amplitudes and latencies is presented in the left (bottom) of the same figure.

**Figure 6:**
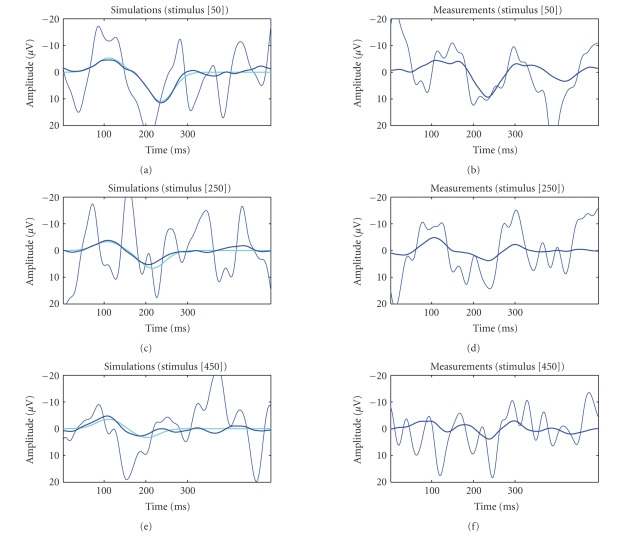
Representative single-trial estimates based on Kalman smoother algorithm. Estimates for the simulations (left) and for the real measurements (right) (standard tones and N100/P200 complex after ocular artifact correction by ICA). Measurements and noisy simulations (dark thin), noise-free simulations (light bold), and estimates (dark bold).

Some representative individual single-trial estimates are presented in Figure 6 for the simulations (left) and real EP measurements (right). The estimates for the simulations and the real EP measurements (standard tones and N100/P200 complex) are based on the artifact corrected EEG and Kalman smoother algorithm. The identification of peak potentials from raw measurements can be misleading even in simple simulations (e.g., stimulus number t=50, left). The proposed method produced accurate estimates for the simulations even in very low SNR conditions (e.g., stimulus number t=450, left). This is because we assumed a trend-like variability. The evaluation of the estimates for the real EPs is naturally more difficult. For example, clear N100 and P200 peaks are obtained for stimuli 50 and 250 (right). Though, the identification of peaks is not trivial for stimulus 450 (right). However, it must be noted that the proposed method does not make assumptions for the number of peaks and their exact form. This information is obtained from the estimated signal subspace and the included eigenvectors.

In summary, the proposed approach for single-trial dynamical estimation of EPs consists of the following steps. (1) Band-pass filter the selected EEG channels. This has as an effect on the improvement of the quality of the signal subspace. For example, it can reduce high-frequency components, and therefore, it can provide smoother eigenvectors and estimates. (2) Enhance the quality of the signal subspace. If the EEG epochs contain strong artifact contributions, such as eye blinks, an artifact correction method can be applied, for example, ICA. (3) Estimate the data correlation matrix and compute eigenvectors. In the simplest case, a basic artifact correction method based on thresholding of potential values and excluding very noisy single-trial epochs can be applied prior to the computation of the correlation matrix. (4) Select a few dominant eigenvectors to form the observation model for estimation. The estimated signal subspace must be able to model latency changes for different phase-locked EP components. (5) Estimate EP characteristics with Kalman smoother algorithm. The smoothing parameter can be selected by visual inspection of the estimates (EP image plots), and by considering the expected trial-to-trial variability of individual peaks.

## 4. DISCUSSION AND CONCLUSION

We presented a new dynamical estimation method for single-trial EP estimation based on a state-space representation for the trial-to-trial evolution of EP characteristics. The method uses the eigenvalue decomposition of the data correlation matrix for the identification of the state-space model. This is an extension of the method presented in [17], where a generic observation model was used. A few dominant eigenvectors obtained from the ensemble measurements incorporate prior information about shape characteristics and within trials correlations of individual EP peaks. This approach takes also into account individual subject characteristics for estimation. Therefore, the method is applicable for different types of EP experiments as long as dynamical behavior from trial-to-trial could be expected. For a Gaussian basis selection like in [17], someone has to select the number of basis vectors and their width. This is not always trivially easy, since a given wave shape may perform in a different way for every individual peak. Therefore, a benefit of SVD is the rather easy selection of observation model that can take into account shape information about different peaks and individual subject characteristics. However, for very weak EPs a generic observation model may have better performance.

Estimates for the state parameters are obtained with Kalman filter and fixed-interval smoother algorithms. Both share the optimality of Bayesian recursive mean square estimation. The fixed-interval smoothing method estimates better the hidden dynamic changes and reduces greater the noise. Therefore, it should be preferred when all the measurements are available. The same behavior can be shown when other observation models are considered, for example, generic basis vectors as in [17]. Therefore, the present paper introduces the use of Kalman smoother algorithm for dynamical estimation of EPs. The use of the filter is appropriate for online estimation. However, compromises between better tracking capabilities and almost online estimation can be searched in terms of fixed-lag smoothing methods [29].

For the demonstration of the methods we used measurements from an auditory experiment (oddball paradigm). Since the aim was to investigate to performance of the methods when strong artifacts exist, we only considered the standard tone measurements and not the deviant and the P300 target response. For this data set the blink artifacts were more prominent for the standard tones. In addition, the estimates of latency and amplitude of the P200 peak (slower and smaller responses towards the end of the measurements) just show that even in ordinary experiments some dynamic behavior from stimulus to stimulus could be expected. However, the method should be addressed to the study of more specific experimental settings. The investigation of latency or amplitude estimates could, for example, be used to study possible habituation effects due to repetition of stimuli, or to study cognitive changes due to time-varying task difficulty or extra distraction. Latency or amplitude changes of peak potentials can also be used to track changes caused by sedative drugs during anesthesia.

EP measurements are usually made with multiple electrodes providing spatial information for the experiment. This information can be used at least to remove artifacts from the signals. We showed by means of ICA that even when the signal subspace is distorted from characteristic artifacts the method is still able to track changes in EP peak components. This is because in the filtering or smoothing procedure phenomena uncorrelated from trial to trial are largely eliminated. In fact, this is exactly the main advantage of dynamical estimation for single-trial EP analysis. However, accurate artifact removal or further elimination of undesirable brain generated components can enable better quality for the signal subspace and individual channel measurements. Extensions to multichannel measurements could be searched by applying the method to each channel separately. Then the variable signal-to-noise ratio conditions from channel to channel should be considered. Another approach could be to direct introduce spatial information in the state-space model. Such multichannel extensions could be investigated for further development of the method. Finally, the signal subspace method can be extended to multichannel measurements. Then it could, for example, be combined with BSS methods.
